# Ionic Liquid 1-Octyl-3-Methylimidazolium (M8OI) Is Mono-Oxygenated by CYP3A4 and CYP3A5 in Adult Human Liver

**DOI:** 10.3390/jox14030050

**Published:** 2024-07-09

**Authors:** Alistair C. Leitch, Tarek M. Abdelghany, Alex Charlton, Martin Cooke, Matthew C. Wright

**Affiliations:** 1 Translational and Clinical Research Institute, Newcastle University, Newcastle upon Tyne NE2 4AA, UK; 2 Department of Pharmacology and Toxicology, Faculty of Pharmacy, Cairo University, Kasr El-Aini St., Cairo 11562, Egypt; tarek.mamdouh@pharma.cu.edu.eg; 3 Institute of Education in Healthcare and Medical Sciences, School of Medicine, Medical Sciences and Nutrition, University of Aberdeen, Aberdeen AB25 2ZD, UK; 4 School of Natural and Environmental Sciences, Bedson Building, Newcastle University, Newcastle upon Tyne NE1 8QB, UK; alex.charlton@newcastle.ac.uk (A.C.); martin.cooke@newcastle.ac.uk (M.C.)

**Keywords:** C8mim, ionic solvent, cytochrome P450, PBC, methylimidazolium ionic liquids

## Abstract

Environmental sampling around a landfill site in the UK previously identified the methylimidazolium ionic liquid, 1-octyl-3-methylimidazolium (M8OI), in the soil. More recently, M8OI was shown to be detectable in sera from 5/20 PBC patients and 1/10 controls and to be oxidised on the alkyl chain in the human liver. The objective of this study was to examine the metabolism of M8OI in humans in more detail. In human hepatocytes, M8OI was mono-oxygenated to 1-(8-Hydroxyoctyl)-3-methyl-imidazolium (HO8IM) then further oxidised to 1-(7-carboxyheptyl)-3-methyl-1H-imidazol-3-ium (COOH7IM). The addition of ketoconazole—in contrast to a range of other cytochrome P450 inhibitors—blocked M8OI metabolism, suggesting primarily CYP3A-dependent mono-oxygenation of M8OI. Hepatocytes from one donor produced negligible and low levels of HO8IM and COOH7IM, respectively, on incubation with M8OI, when compared to hepatocytes from other donors. This donor had undetectable levels of CYP3A4 protein and low CYP3A enzyme activity. Transcript expression levels for other adult CYP3A isoforms—CYP3A5 and CYP3A43—suggest that a lack of CYP3A4 accounted primarily for this donor’s low rate of M8OI oxidation. Insect cell (supersome) expression of various human CYPs identified CYP3A4 as the most active CYP mediating M8OI mono-oxygenation, followed by CYP3A5. HO8IM and COOH7IM were not toxic to human hepatocytes, in contrast to M8OI, and using a pooled preparation of human hepatocytes from five donors, ketoconazole potentiated M8OI toxicity. These data demonstrate that CYP3A initiates the mono-oxygenation and detoxification of M8OI in adult human livers and that CYP3A4 likely plays a major role in this process.

## 1. Introduction

Ionic liquids are a structurally diverse range of chemicals. However, they are all salts, have low volatility and are often liquids at ambient temperatures [1]. These physical characteristics make them useful solvents, and because they are not volatile, they are often proposed as environmentally friendly alternatives to traditional solvents [2]. 

**Figure 1:**
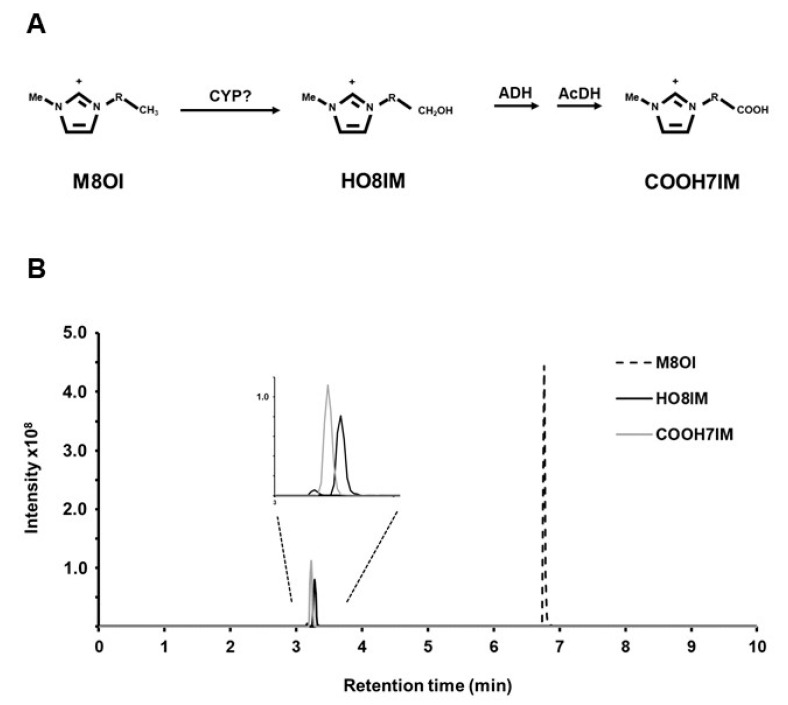

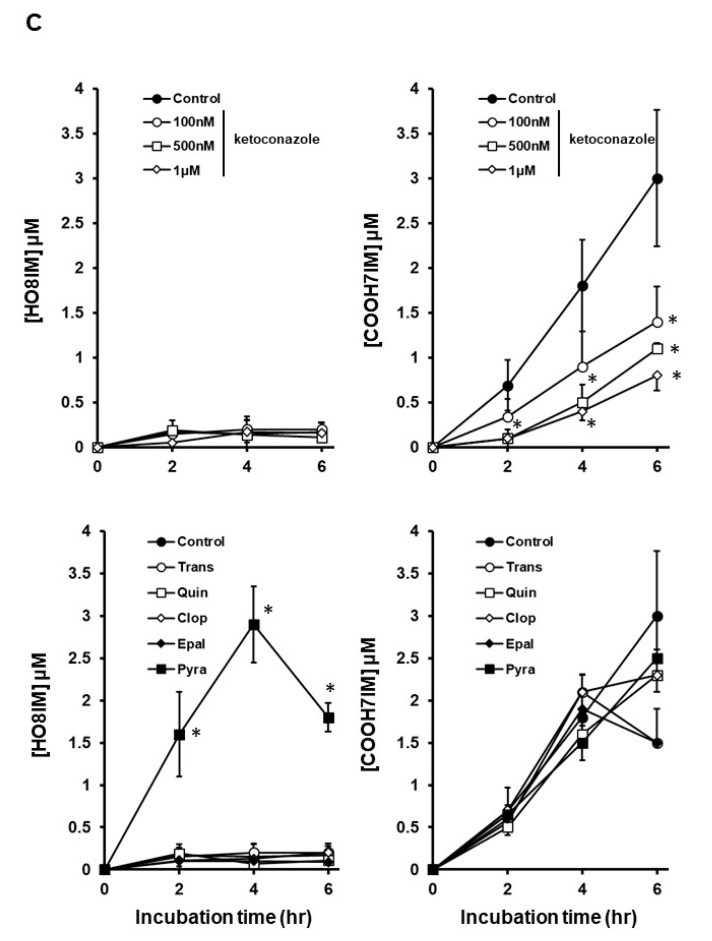
The metabolism of M8OI in the human liver. (**A**) The proposed metabolic pathway of M8OI metabolism. CYP, cytochrome P450 based on this inhibition by the CYP inhibitors SKF525a and metyrapone, as reported in [10]; ADH, alcohol dehydrogenase; AcDH, acetaldehyde dehydrogenase, based on inhibition by the ADH inhibitor 4-methyl pyrazole and AcDH inhibitor disulfiram, as reported in [10]. Note, R = -(CH_2_)_7_-. (**B**) A typical LC-MS chromatogram from human hepatocytes (NHL5) during incubation with 10 µM M8OI demonstrating the production of HO8IM and COOH7IM (with similar retention times but identified and quantified based on their different fragmentation patterns); MRM transitions for M8OI, HO8IM and COOH7IM were m/z 195.2 -> 83.1, *m*/*z* 211.2 -> 83.1 and *m*/*z* 225.2 -> 83.1. (**C**) The time course for the production of HO8IM and COOH7IM in human hepatocyte (NHL5) incubation with 10 µM M8OI with or without the co-incubation of the indicated CYP inhibitor (see Table 1 for details). Data are the mean and SD of 3 separate cultures from the same hepatocyte donor. * *p* > 0.95.

Methylimidazolium ionic liquids (MILs) are composed of a methylimidazolium cation in combination with a variety of different anions (from Cl^−^ to more complex anions such as bis-[trifluoromethanesulfonyl]-imide). MILs are man-made and their use is currently restricted to industrial processes [2,3]. Accordingly, their technological uses and use levels are generally kept confidential for commercial reasons. The length of the alkyl chain varies in MILs (for general structure, see Figure 1A), with those containing a short alkyl chain (i.e., 2C and 4C variants) used at the highest levels [3]. Longer-chain variants show increasing toxicity [4] and are probably used only to a limited extent [3]. 

1-octyl-3-methylimidazolium chloride (M8OI) is a longer chain MIL and recent research traced toxicity in soil samples to the presence of the M8OI cation [5]. Subsequent investigations indicated that M8OI was toxic to a variety of mammalian cells in vitro through the disruption of mitochondrial function [4] and in vivo (with the kidney being a target organ) [6,7]. Adverse cardiac effects have also been identified [8]. M8OI has also been shown to activate the human oestrogen receptor in vitro [9]. 

M8OI is subject to limited metabolism in human and rodents. It is initially mono-oxygenated to 1-(8-hydroxyoctyl)-3-methyl-imidazolium (HO8IM), most likely by cytochrome P450s (CYPs) based on broad-spectrum CYP inhibitor studies in human hepatocytes [10]. HO8IM then undergoes sequential oxidation to 1-(7-carboxyheptyl)-3-methyl-1H-imidazol-3-ium (COOH7IM), likely by alcohol (ADH) and acetaldehyde dehydrogenases (AcDH), based again on broad-spectrum inhibitor studies [10]. No other additional metabolic conversion has been detected to any substantial extent, suggesting that M8OI may be resistant to extensive degradation.

Our interest in M8OI is based around identifying potential xenobiotic triggers for the autoimmune liver disease primary biliary cholangitis (PBC). This is because COOH7IM can be inserted enzymatically in place of lipoic acid in the major PBC auto-antigen [5]. Furthermore, M8OI has additional adverse hepatic effects that could potentiate a PBC response, including cholangiopathic effects (induction of liver progenitor cell apoptosis) and cholestatic properties [5]. In this respect, it is notable that M8OI has been detected in sera from 5/20 PBC patients and 1/10 controls [10]. Given that M8OI use is restricted to industrial processes [3], its presence in the environment and the human population is unexpected. Given the limitations in available toxicological data for M8OI and related MILs [3], exposure to the human population should be negligible. Given its presence in the environment, the main route of exposure in the human population is likely oral through diet. Dermal and inhalation (e.g., via aerosolised liquid droplets) routes of exposure may also be a risk in occupational exposure. 

Understanding the metabolism of xenobiotics is a fundamental component in the studies needed to understand the toxicity and potential variation in susceptibility in the human population. The objective of this study is to examine the metabolism of M8OI in humans in more detail. In this study, we report hepatocytes from an individual with a limited capacity to metabolise M8OI and found that the hepatocytes from this donor lacked CYP3A4 protein expression. We then demonstrate that recombinant CYP3A4—followed by the closely related CYP3A5—was active in the hydroxylation of M8OI. We further demonstrate that the conversion of M8OI to COOH7IM is a detoxication pathway since the inhibition of its metabolism increases M8OI toxicity in cultured hepatocytes pooled from five donors.

## 2. Materials and Methods

### 2.1. Materials

HO8IM and COOH7IM were custom synthesised by Fountainbridge (Edinburgh, UK) with purity (99.7 and 99.3%, respectively) and chemical structures confirmed by HPLC, mass spectrometry and NMR techniques, as previously published [5,10]. Supersomes were obtained from Corning, Loughborough, UK. The following antibodies were used in Western blotting and/or immunohistochemistry: anti-CYP3A4 (Abcam, ab124921), anti-CYP2E1 (Abcam, ab28146), anti-CPSI (Abcam, ab3682), anti-POR (Santa Cruz, sc-13984) and anti-β-actin (Merck, A1978). M8OI (>96% purity) and all other chemicals were purchased from Sigma (Poole, UK), unless otherwise stated.

### 2.2. Human Hepatocyte Isolation and Culture

Hepatocytes were isolated by using a 2-step collagenase perfusion procedure as outlined in [24], with specific approval from a local research ethics committee (see Institutional Review Board Statement). Livers were included in this study if they had initially been retrieved for transplantation (lacked moderate to severe steatosis based on either visual appearance or histological evidence; lacked donor comorbidities considered to make a significant impact on graft survival) but were subsequently declined by all seven UK liver centres. Consent from the donor’s next of kin was required for inclusion into an organ reconditioning research study prior to hepatocyte isolation. In brief, livers were flushed and perfused in situ with ice-cooled University of Wisconsin preservation solution (ViaSpan), then surgically removed from donors with cannulation of the hepatic artery, portal vein, inferior vena cavae and common bile duct in place. Livers were transported on ice prior to perfusing the organ with isotonic (0.9% *w*/*v*) sterile NaCl to remove the preservation solution. The liver was then connected to a perfusion circuit consisting of CARMEDA^®^ BioActive Surface heparin-tubing, a Medtronic Bio-Console 560 Speed Pump Controller and a Hirtz Hico-Variotherm 555 heater–cooler. Perfusion was then raised to 37 °C with oxygenated human erythrocyte-based perfusate (3 units of type-specific human packed red blood cells, 10,000 units of unfractionated heparin, 10 mL of 10% (*w*/*v*) calcium gluconate, 500 mL of Isoplex 4% (*w*/*v*) (succinylated gelatin) solution, 8.4% (*w*/*v*) sodium bicarbonate, approximate total initial volume of 1.25–1.5 L) to recondition the organ. The circuit included dual perfusion of the graft via the hepatic artery and portal vein in a closed system (hepatic artery, portal vein, bottom-opening inferior vein cava and common bile duct are cannulated). Perfusion pressures in the hepatic artery and portal vein were maintained at 70 mm and 5 mm, respectively, using pinch valves applied to the inflow tubing of the portal vein. Heparin, Flolan (epoprostenol sodium), Actrapid (recombinant human insulin), Synthamin 9 (5.5% amino acids infusion) and Cernevit (multivitamins for infusion) were injected into the perfusion circuit when required. Blood gas analysis was employed to monitor perfusate pO2 and pH with additions of sodium bicarbonate and oxygen used to maintain a perfusate pH > 7.2 and pO2 > 15 kPA respectively. After reconditioning (6–24 h), the liver was surgically split to allow for fully cannulated perfusion (at 150 mL/min @ 37 °C) of the left lateral segment of the liver. Human hepatocytes were then isolated from this segment following a 2-step collagenase perfusion procedure (with collagenase step recirculated until digestion, typically 20–30 min). When the liver appeared digested, it was removed from the perfusion and placed in a sterile dish and the capsule was torn to release the digested cell suspension. The suspension was recovered using a sterile pipette and filtered through sterile 100 µm nylon bolting cloth to remove undigested tissue. The collected filtered suspension was then subjected to repeated centrifugation at room temperature (50 g, 3 min) with pelleted cells re-suspended in Ca/Mg-free Hank’s balanced salt solution (0.14 M NaCl, 5.4 mM KCl, 0.34 mM Na_2_HP0_4_.7H_2_0, 0.44 mM KH_2_P0_4_, 5.6 mM D-glucose and 15.7 mM NaHC0_3_). After 3–5 centrifugation steps, the cell suspension was re-suspended in Williams medium E supplemented with 10% (*v*/*v*) FCS, 80 µg/mL penicillin, 80 µg/mL streptomycin, 10 nM dexamethasone and 1 µg/mL insulin. Cell viability was determined by trypan blue exclusion and cells were seeded at a density of 10 million cells/7 mL of culture medium on collagen-coated (95–98% type I) plates in a humidified atmosphere of 5% CO_2_/95% air at 37 °C. After an overnight culture period, the medium was aspirated, the cells were washed 3 times with sterile PBS (137 mM NaCl, 2.7 mM KCl, 10 mM phosphate pH 7.4) and subsequently cultured in fresh medium without serum, with a daily medium change thereafter. The liver doners were as follows: NHL5, 50-year-old male; Liver A, 23-year-old male; Liver B, 64-year-old female.

Cryopreserved hepatocytes (Xenotech HPCH05+; Lot No. 2110283; for details of donors and CYP probe substrate activities, see Supp. Figure S1) pooled from 5 donors were also purchased through Tebubio (France). Hepatocytes were defrosted, seeded onto 96-well collagen-coated plates (Greiner Bio-One) and cultured following the supplier’s instructions and using the supplier’s recommended media (OptiThawTM, OptiPlateTM and OptiCultureTM supplemented with 0.25 mg/mL OptiMatrixTM). After an overnight recovery period, the media were removed and hepatocytes were challenged with M8OI in OptiCultureTM medium. Additional compounds were added from a 1000-fold DMSO solvated stock with 0.1% *v*/*v* DMSO added to controls.

### 2.3. Immunohistochemistry

First, 1 cm^3^ pieces of liver tissue were removed from the donor organ after normothermic machine perfusion and fixed in 10% (*v*/*v*) formalin in PBS. The following day, the tissues were transferred to ethanol prior to standard histopathology processing and embedding in wax. Sections were stained with haematoxylin and eosin or immunostained as previously described [25]. For all immunostaining, sodium citrate antigen retrieval was used to expose epitopes, and primary antibodies were used at a 1:250 dilution. Di-amino benzidine (Dako, Ely, UK) was used to visualise secondary antibody binding to primary antibodies (anti-mouse/HRP or anti rabbit/HRP, 1:250 dilution, Dako). All sections were counterstained with haematoxylin prior to mounting.

### 2.4. Gene Expression

RNA was isolated from human hepatocytes after overnight recovery using TRI Reagent^®^ (Sigma, Poole, UK) and following the manufacturer’s protocol. qRT-PCR was performed on total RNA using Taqman probes and an Applied Biosystems7500 Real-Time PCR machine, as previously described [26], and validated Taqman primer kits purchased from Thermo Fischer Scientific [18S rRNA, Hs99999901_s1; CYP1A1, Hs01054796_g1; CYP1A2, Hs00167927_m1; CYP2E1, Hs00559367_m1; CYP3A4, Hs00604506_m1; CYP3A5, Hs02511768_s1; CYP3A43, Hs01119078_mH; POR, Hs01016332_m1].

Western blotting was performed as previously described [26]. In brief, protein samples were separated by SDS-PAGE (typically 4% stacking; 9% separating gels) under reducing conditions using the MiniP2 Bio-Rad electrophoresis apparatus. Protein was transferred onto nitrocellulose and membranes were blocked (to prevent non-specific binding of antibodies) overnight with TBS buffer (20 mM Tris, 200 mM NaCl, pH 7.4) containing 3% (*w*/*v*) milk protein and 0.3% (*w*/*v*) Tween 20. After incubation with primary antibodies [rabbit monoclonal anti-CYP3A4 (Abcam, ab124921); rabbit polyclonal anti-CYP2E1 (Abcam, ab28146); rabbit polyclonal anti-CPSI (Abcam, ab3682); rabbit polyclonal anti-POR (Santa Cruz, sc-13984); or mouse monoclonal anti-β-actin (Merck, A1978)] in TBS supplemented with 0.3% (*w*/*v*) milk protein and 0.05% (*v*/*v*) Tween 20, blots were incubated with the appropriate horseradish peroxidase-conjugated anti-IgG antibody, followed by ECL detection and X-ray film exposure.

### 2.5. CYP3A Activity

CYP3A activity was determined in hepatocytes using a Luciferin PPXE (#V8912, DMSO tolerant) assay kit purchased from Promega according to the manufacturer’s instructions except that 2 mM NADPH was used as a cofactor. Cell extracts were prepared in 100 mM KPO_4_ buffer at pH 7.4 and protein contents were determined using the Lowry method.

### 2.6. M8OI Incubation with Hepatocytes

The culture medium was removed and the cells were washed 3 times in PBS. Hepatocytes were then incubated for up to 24 h with 10 µM M8OI in 0.10 M NaCl, 5.4 mM KCl, 0.34 mM Na_2_HPO_4_ 12H_2_O, 0.44 mM KH_2_PO_4_, 20 mM glucose, 1 mM CaCl_2_, 40 mM NaHCO_3_, 4 mM glutamine, 100 µM L-alanine, 100 µM L-asparagine, 100 µM L-aspartic acid, 100 µM L-glutamic Acid, 100 µM glycine, 100 µM L-proline and 100 µM L-serine (pH 7.4 when gassed with 5% CO_2_ in air) with or without the addition of CYP inhibitors (see Table 1). Samples of the medium were removed at various times and centrifuged (10,000 rpm at 4 °C for 20 s) to remove any cellular material prior to acidification with 0.1 volumes of 1% phosphoric acid. Samples were then snap-frozen at −80 °C until analysis.

### 2.7. M8OI Metabolism in Supersomes

M8OI metabolism was examined with individual CYPs in membranes (supersomes) isolated from insect cells infected with recombinant baculovirus encoding individual CYPs of interest (Corning, UK). In all cases, supersomes contained CYP reductase and the cytochrome B_5_. Incubations were performed for up to 3 h in 100 mM Tris buffer at pH 7.5 containing 1 mM NADPH and 50 μM M8OI. Reactions were pre-incubated at 37 °C for 5 min before the addition of 20 pmol CYP/mL. Identical incubations except for the addition of CYP (i.e., supersomes) were included as a control. Reactions were halted by the addition of 50 µL of the reaction mixture to 50 µL of ice-cooled acetonitrile. Samples were then subjected to centrifugation at 10,000 rpm at 4 °C for 3 min. Supernatants were removed and stored at −20 °C until analysis.

### 2.8. M8OI and Metabolite Determinations

M8OI and M8OI metabolites (HO8IM, COOH7IM) were quantified by standard multiple reaction monitoring (MRM) techniques using a Waters Xevo TQ-S Triple Quadrupole Mass-Spectrometer coupled to a Waters BEH-C18 (100 × 2.1 mm; 1.7 μm particle size) column. Separation was achieved by gradient elution with (A) 0.1% formic acid in water and (B) 0.1% formic acid in acetonitrile at a flow rate of 250 μL/min [5% B at t = 0; 30% B at t = 5 min; 95% B at t = 8 min; 95% B at t = 9 min; 5% B at t = 9.5 min; 5% B at t = 12 min]. Metabolites (1 μL injection volume per sample) were separated at 40 °C using an ACE Excel 3 C18-PFP (150 mm × 2.1 mm; 3 μm particle size) chromatography column equipped with an ACE Excel UPLC Pre-Column filter guard column. MRM transitions for M8OI, HO8IM and COOH7IM were m/z 195.2 -> 83.1, *m*/*z* 211.2 -> 83.1 and *m*/*z* 225.2 -> 83.1, respectively, with a collision energy of 20 eV.

### 2.9. Thiazolyl Blue Tetrazolium Bromide Assay

Thiazolyl blue tetrazolium bromide (MTT) reduction was determined as previously described [27]. In brief, MTT reduction was determined following the addition of 0.5 mg MTT/mL medium to cultured hepatocytes and further incubation for 1 h at 37 °C in the humidified incubator used for culturing. The medium was then removed and replaced with a fixed volume of isopropanol for 10–15 min to liberate the intracellular insoluble reduced MTT product. A fixed volume of the isopropanol was then removed, and absorbance was determined at 570 nm (with background absorbance at 690 nm also determined and the reading subtracted from the reading at 570 nm). The results are expressed as the percentage of absorbance relative to vehicle-treated cells. M8OI does not directly inhibit MTT reduction [27] and is used as a proxy for cell viability.

### 2.10. Statistical Analyses

For testing the difference between one independent variable and one variation in treatment, Student’s *t*-test was used. For a study with 3 or more conditions with 1 independent variable, a one-way ANOVA was used, followed, when required, by a Bonferroni post-hoc test. In all cases, significant difference (2-tailed) was judged at the *p* < 0.05 level.

## 3. Results

### 3.1. M8OI Metabolism by Human Hepatocytes Is Potently Inhibited by Ketoconazole in a Dose-Dependent Manner

Previous investigations in human hepatocytes identified that broad-spectrum relatively non-specific CYP inhibitors (SKF525a, metyrapone) inhibited M8OI metabolism [10]. The metabolism of M8OI was therefore examined in human hepatocytes in the presence of a range of relatively specific CYP inhibitors (see Table 1).

Figure 1B demonstrates a typical LC-MS chromatogram from human hepatocytes incubated with M8OI. Figure 1C demonstrates that HO8IM was barely detectable in hepatocyte cultures, except in the presence of pyrazole, which is a CYP2E1 and ADH inhibitor [19,20]. This accumulation of HO8IM was likely due to the inhibition of ADH and suggests—under normal circumstances (i.e., in the presence of uninhibited ADH)—there is rapid conversion of HO8IM to COOH7IM. Accordingly, the effects on COOH7IM production were considered indicative of CYP inhibition despite the metabolite not being a direct CYP-mediated product. Figure 1C suggests that CYP2A6, CYP2B6, CYP2D6, CYP2E1 and CYP4A11 do not make a major contribution to the hydroxylation of M8OI in hepatocytes based on the limited effects of their inhibitors. In contrast, the addition of low (10^−7^–10^−6^ M) concentrations of ketoconazole resulted in a dose-dependent statistically significant decrease in COOH7IM production, suggesting that CYP3As make the major contribution to M8OI mono-oxygenation in the human liver.

### 3.2. Metabolism of M8OI Is Significantly Reduced in Hepatocytes Isolated from Donor Expressing Low Levels of CYP3A4

**Figure 2:**
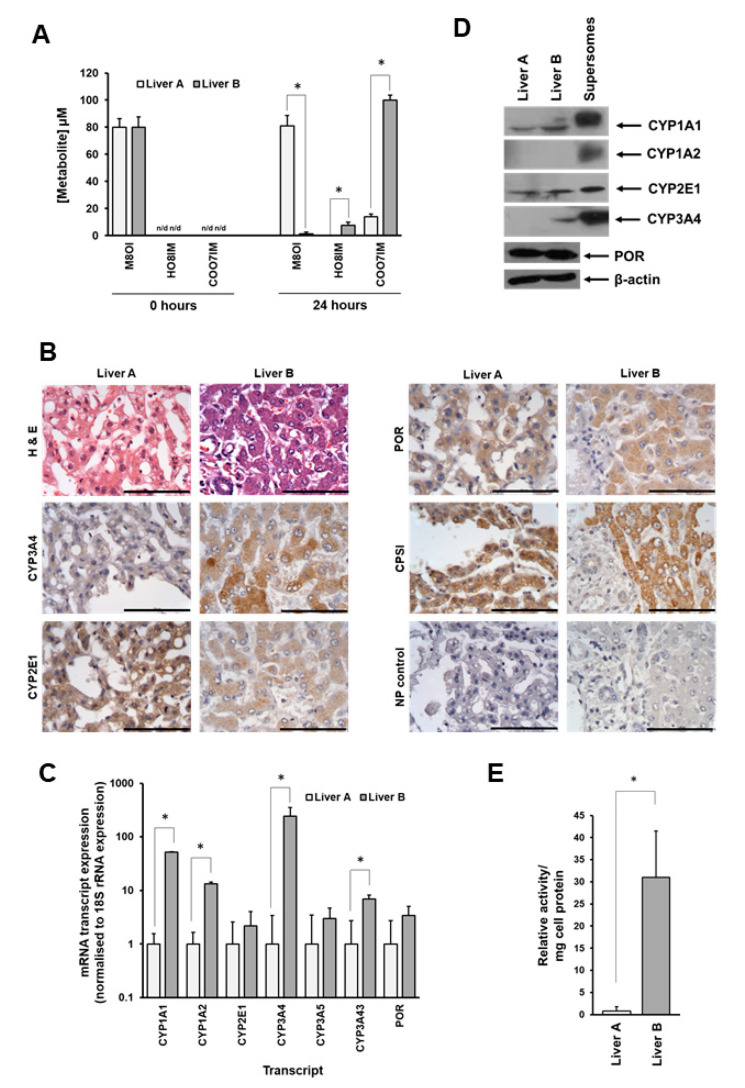
Concentrations of M8OI and its metabolites in human hepatocyte cultures. (**A**) Hepatocytes were isolated from Liver A or Liver B and cultured as outlined in the Materials and Methods Section. Hepatocytes were incubated with 100 μM M8OI and the concentrations of M8OI, HO8IM and COOH7IM were determined in triplicate cultures directly (within 5 min) after addition and after 24 h. Concentrations were determined by reference to authentic standards. * *p* > 0.95. (**B**) H & E or immunohistochemical staining of liver sections from Liver A and Liver B for the indicated protein. For CYPs, a centrilobular region of the lobule is shown. POR, cytochrome P450 oxidoreductase; CPSI, carbamylphosphate synthase I (a liver-specific protein). Bar = 100 μm. (**C**) qRT-PCR for the indicated transcripts in hepatocytes isolated from Liver A and Liver B. RNA was isolated from 3 aliquots of cells and transcript levels were determined after normalisation to 18S rRNA levels. Normalised expression for each transcript was then expressed relative to the normalised level present in Liver A. * *p* > 0.95. (**D**) Western blot for the indicated protein from hepatocytes isolated from the indicated liver. Note: liver extracts, 20 µg protein/well; supersomes, 2 µg protein/well. (**E**) Luciferin PPXE activities. Data are the mean and SD of 3 determinations from each extract and are normalised to total protein levels. * *p* > 0.95.

The metabolism of M8OI was examined in hepatocyte cultures isolated from several donors through the addition of 10 µM M8OI to the incubation medium, as outlined in the Materials and Methods Section. In all but one preparation, M8OI was extensively metabolised to COOH7IM (Figure 1A–C) such that by 24 h, incubation medium M8OI concentrations were markedly depleted, accompanied by near-complete conversion to COOH7IM and low levels of HO8IM, as seen with hepatocytes isolated from Liver B (Figure 2A). Liver A, however, did not give rise to a statistically significant depletion of incubation medium M8OI and showed only low levels of COOH7IM production (Figure 2A). Based on an MTT assay performed after 24 h of exposure, there was no toxicity observed in both Liver A and B hepatocytes at the concentration of M8OI used in these metabolism studies (Supp. Figure S1).

An examination of the donor livers indicated that the major expressed hepatic cytochrome P450—CYP3A4—was expressed at low levels in hepatocytes in intact Liver A, whereas CYP2E1 was expressed and readily detectable in both liver A and Liver B as determined by immunohistochemistry (Figure 2B). This apparent marked disparity in CYP3A4 expression between donors was supported by a more than 200-fold lower expression of CYP3A4 mRNA transcript (Figure 2C); there were also undetectable levels of CYP3A4 protein (Figure 2D) and relatively low levels of CYP3A enzyme activity in hepatocytes isolated from Liver A compared to Liver B (Figure 2E).

These data suggest that the reduced rate of M8OI metabolism in hepatocytes from Liver A was due to the low levels of expression of CYP3A4.

### 3.3. Supersome Recombinant CYP3A4 Mono-Oxygenated M8OI to form HO8IM and Two Other Hydroxylated Metabolites

**Figure 3:**
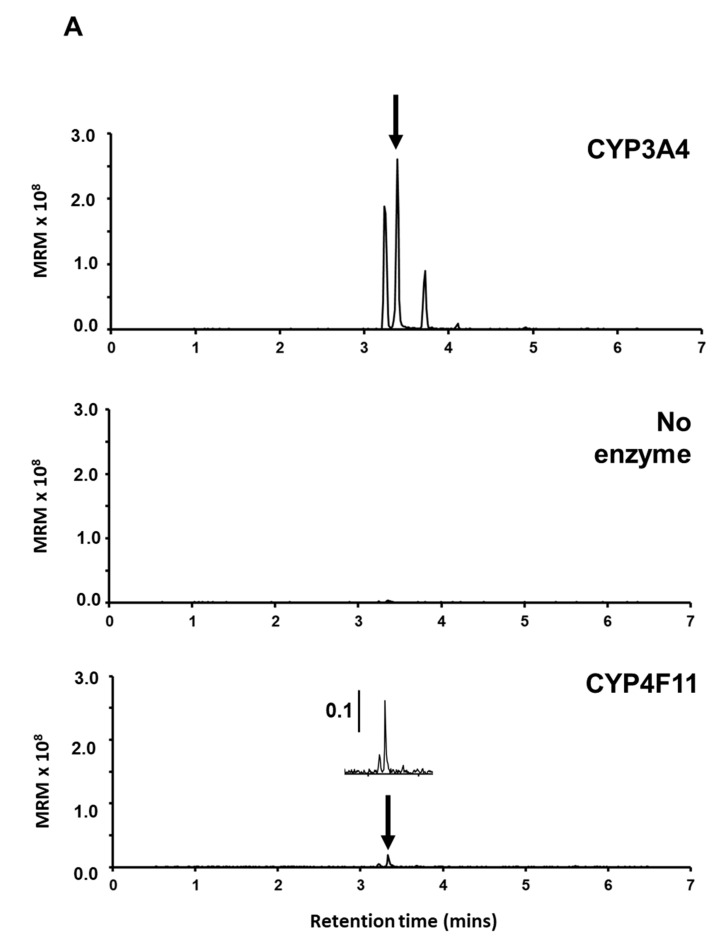

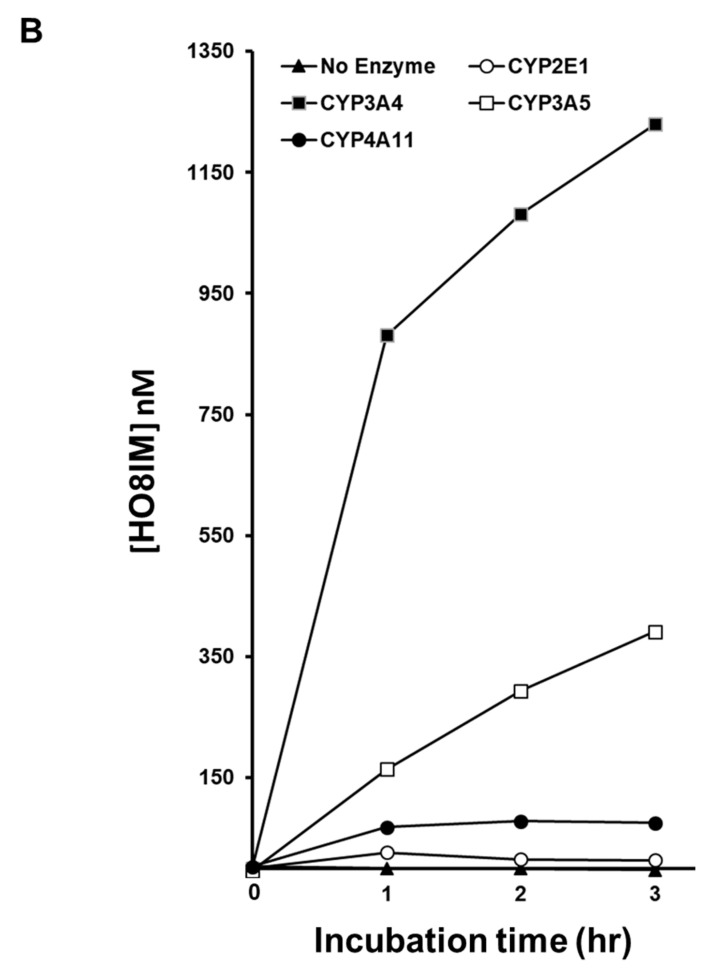
M8OI metabolism with individual CYPs in membranes (supersomes) isolated from insect cells infected with recombinant CYP baculovirus. (**A**) A typical LC-MS chromatogram from the indicated supersome incubations with M8OI demonstrating the production of HO8IM; MRM transition *m*/*z* 211.2 -> 83.1. The identity of HO8IM was determined using an authentic standard (also seen with hepatocyte incubations) and is indicated by arrows. (**B**) The time course for the production of HO8IM with the indicated recombinant CYP. HO8IM concentrations were determined using authentic HO8IM as the standard. Note that in all cases, supersomes contained additionally both CYP reductase and cytochrome B5. The results are typical of at least 3 separate determinations of the same batch of supersome CYP.

Using recombinant CYP3A4 expressed in insect cell membrane extracts (supersomes), Figure 3A demonstrates that M8OI is mono-oxygenated to HO8IM. However, in contrast to hepatocytes, an additional two other closely migrating peaks with identical MRM masses to HO8IM and similar abundance were observed. These peaks are likely associated with hydroxylations elsewhere on the alkyl chain. However, in the absence of an authentic standard other than for HO8IM, the position and identity of the metabolite responsible for these peaks could not be determined. The production of HO8IM was not seen in the absence of enzymes (Figure 3A).

Quantitation of HO8IM production over time demonstrates that CYP3A4 was the most active of the CYPs examined (Figure 3B). CYP3A5 was also moderately active, whereas CYP2E1 and CYP4A11 mono-oxygenated M8OI at relatively slow rates (Figure 3B).

No production of COOH7IM from M8OI was observed in any supersome experiments, supporting the suggestion that this latter step (i.e., HO8IM -> COOH7IM) is mediated by dehydrogenases. Although CYPs have been shown to mediate the oxidation of substrates (e.g., ethanol to acetaldehyde [28,29], this does not appear to be the case at all with M8OI and the CYPs chosen in this study.

### 3.4. The Oxidation of M8OI Represents a Detoxification Pathway in Human Hepatocytes

**Figure 4:**
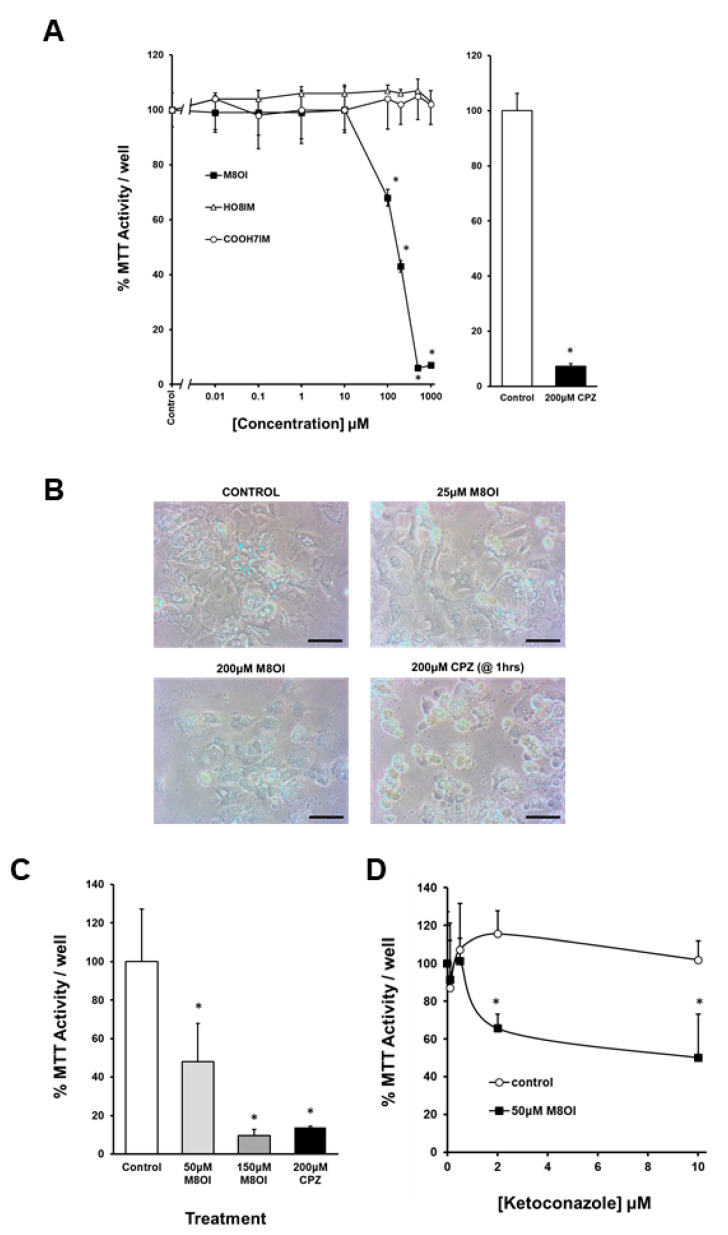
The toxicity of M8OI in human hepatocytes is potentiated by the inhibition of its metabolism. (**A**) Left panel, human hepatocytes (NHL5) were treated with increasing concentrations of either M8OI, HO8IM or COOH7IM for 24 h, or right panel, 200 μM chlorpromazine (CPZ) for 1 h; control hepatocytes were treated with a 0.1% *v*/*v* DMSO vehicle. For MTT reduction, the data are the mean and SD of 4 separate determinations from the same donor. * *p* > 0.95 using Student’s *t*-test. (**B**) Photomicrographs of hepatocytes treated for 24 h as indicated, unless otherwise indicated. Bar = 100 μm. (**C**) Cryopreserved cultured hepatocytes treated for 24 h as indicated (except CPZ: 1 h). For MTT reduction, the data are the mean and SD of 4 separate determinations. Shown is the statistically significantly different % MTT activity versus the control (2-tailed, *p* < 0.05 level) using Student’s *t*-test. (**D**) Cryopreserved cultured hepatocytes treated for 24 h with or without 50 μM M8OI and increasing concentrations of ketoconazole. For MTT reduction, the data are the mean and SD of 4 separate determinations. * Statistically significantly different % MTT activity versus equivalent ketoconazole-free cultures (2-tailed, *p* < 0.05 level) using Student’s *t*-test. Note: chlorpromazine leads to hepatocyte death via necrosis [30].

Hepatocytes from donor NHL5 (Figure 4A) were typical of several investigations with different donors and demonstrate that M8OI is toxic to human hepatocytes with an EC_50%_ between 100 and 200 μM, with a lack of toxicity seen with the HO8IM and COOH7IM metabolites up to 1 mM over 24 h. Morphological examination suggests vacuole formation in hepatocytes (Figure 4B) typically seen in B-13 cells and associated with mitochondrial dysregulation [5].

The relatively low sensitivity of human hepatocytes to M8OI compared to B-13 cells is likely to be associated, at least in part, with M8OI metabolism in human hepatocytes. To further test the hypothesis that the conversion of M8OI to COOH7IM is a detoxification pathway, hepatocytes were treated with M8OI and the effect of a block in metabolism was examined. In this case, hepatocytes pooled from five donors were used in order to identify a likely general effect in humans. Using this approach, cryopreserved hepatocytes were the only practicable choice, necessitating alternative seeding and maintenance protocols. Figure 4C indicates that these pooled hepatocytes were slightly more sensitive than hepatocytes isolated and cultured in-house. However, a dose-dependent increase in toxicity was still observed with M8OI (Figure 4C). Ketoconazole was not toxic to these hepatocytes, yet its co-incubation with 50 µM M8OI increased M8OI toxicity in a ketoconazole-dependent manner (Figure 4D). These data therefore support the concept that the metabolic conversion of M8OI to both HO8IM and COOH7IM represents detoxification steps in terms of its acute toxicity.

## 4. Discussion

The objective of this study was to examine the metabolism of M8OI in adult human livers in more detail, specifically to identify the isoform of CYP that is most prominent in its mono-oxygenation.

Ketoconazole is widely recognised as a relatively specific inhibitor of CYP3A isoforms at sub-micromolar concentrations [22], and given its potent inhibitory effect on M8OI metabolism, CYP3A isoforms are likely to be the major players in M8OI conversion to HO8IM in intact cells. There are four CYP3A genes in humans: CYP3A4, CYP3A5, CYP3A7 and CYP3A43. CYP3A7 expression, once thought to be an enzyme exclusive to foetal livers, has more recently been identified in neonates and developing infants as old as 24 months post-gestational age [31]. Given its negligible expression postnatally, it is unlikely that CYP3A7 plays any role in M8OI metabolism in our studies. Little is known about CYP3A43 [32], and a supersome preparation for this isoform is not available. A role, if any, of this isoform in M8OI metabolism therefore cannot currently be determined. CYP3A4 is generally considered the major expressed human liver CYP and proposed to be involved in the metabolism of between 30 and 64% of clinically prescribed drugs [33]. Given these facts and that supersomes containing CYP3A4 are the most active in the metabolism of M8OI, it is likely that in adult humans, CYP3A4 would make a major contribution to the metabolism of M8OI. However, CYP3A5 may also make a minor contribution to this metabolism.

Interestingly, supersome incubations result in the production of several hydroxylated metabolites not previously noted in cell extracts. There is evidence for the production of a low level of one of these in human hepatocytes (see Figure 1B). However, it is likely that these additional metabolites in supersome preparations appear because the products are not subsequently oxidised by the dehydrogenases present and are relatively highly active in hepatocytes and other cells. The supersome component of this study suggests that CYP3A4 and CYP3A5 likely make major contributions to the metabolism of M8OI in the human liver. However, CYPs are highly polymorphic and supersome preparations cannot represent every functional CYP gene variant, limiting the certainty of this conclusion. A prominent role for CYP3A4 is however supported by the slow rate of M8OI metabolism seen in hepatocytes isolated from a donor with negligible CYP3A4. This is somewhat of a chance determination since CYP3A4 is a major hepatic form and finding individuals lacking this enzyme is highly rare. Given the paucity of donor liver tissue available, the cost and difficulties of isolating viable cells and the de-differentiation of hepatocytes in vitro (meaning that retrospective confirmatory experiments cannot be performed), there always remains a limitation on studies performed with human hepatocytes. However, as a qualitative study, this study clearly identifies for the first time the isoforms of CYP that mediate the metabolism of M8OI but cannot address the quantitative assessment of M8OI in the general population with any certainty.

There is likely also some contribution from other less active CYPs to the mono-oxygenation of M8OI in hepatocytes. This could explain the appearance of COOH7IM in hepatocyte incubations of hepatocytes lacking CYP3A4, in that low levels of HO8IM production by other CYPs would be rapidly converted to COOH7IM by dehydrogenases. Fatty acids are known to undergo mono-oxygenation on the ω carbon as part of a process of increasing fatty-acid oxidation [34]. This metabolic pathway includes subsequent oxidation of the hydroxylated carbon to a carboxylic acid, as seen with M8OI. Accordingly, CYP4A11 was included as a potential CYP mediating M8OI mono-oxygenation. However, the data in this paper indicate that this isoform was relatively slow in mediating the mono-oxygenation of M8OI.

Our primary interest in M8OI is based around identifying potential xenobiotic triggers for the autoimmune liver disease PBC. As previously stated, COOH7IM can be inserted enzymatically in place of lipoic acid in the major PBC auto-antigen pyruvate dehydrogenase E2 subunit [5]. The data in this paper suggest that if such a mechanism should occur in vivo, then the process may be significantly influenced by hepatic CYP3A4 and, potentially to some extent, CYP3A5 expression. Interestingly, PBC is widely reported to be predominantly seen in post-menopausal women, with female predominance ranging between around 80 and 90% [35,36,37,38]. Both CYP3A4 and CYP3A5 isoforms are more highly expressed in women than in men [39,40].

Previous research on cell lines has shown that M8OI is toxic to cells through its inhibition of mitochondrial oxidative phosphorylation and induction of apoptosis [4]. For example, using MTT as a proxy for cell viability, the EC50% for M8OI toxicity in B-13 cells was shown to be 6.4 ± 0.72 μM [4], which do not appreciably metabolise M8OI. The data in this paper suggest a similar mechanism of toxicity but reduced sensitivity, likely predominantly associated with the metabolism of M8OI in human hepatocytes to less toxic metabolites.

However, although there may be a plausible pathway and hazard with respect to M8OI and the triggering of PBC, there are very limited data on population exposure. Therefore, the suggestion that M8OI represents a risk for triggering PBC remains hypothetical. However, the data in this paper demonstrate for the first time that CYP3A initiates the mono-oxygenation and detoxification of M8OI in the human liver and that CYP3A4 likely plays a major role in this process.

## Figures and Tables

**Table 1:** CYP inhibitors used in M8OI metabolism studies with human hepatocytes.

CYP	Inhibitor Concentration	References/Comments
CYP2A6	Tranylcypromine 5 μM	The (R)-enantiomer in human liver microsomes inhibits CYP2A6 at Ki values of 0.04–0.2 μM [11,12,13].
CYP2B6	Clopidogrel 5 μM	Clopidogrel inhibits CYP2B6 at IC50 values of 0.0146–0.046 μM [14,15,16].
CYP2D6	Quinidine 5 μM	This inhibits CYP2D6 at Ki values of 0.03–0.4 μM [17,18].
CYP2E1	Pyrazole 2 mM	This inhibits CYP2E1 (Ki of 35.5 μM). It also inhibits ADH [19,20].
CYP3A4/3A5/3A7/3A43	Ketoconazole 100 nM–1 μM	Using recombinant enzymes, Ki values of 0.0267 and 0.109 μM for CYP3A4 and CYP3A5, respectively [21]. Ketoconazole is a very selective inhibitor of CYP3A when used at sub-micromolar concentrations [22].
CYP4A11	Epalrestat 5 μM	Using recombinant enzymes, a Ki value of 1.82 μM for CYP4A11. The most potent inhibition out of 17 other recombinant CYPs [23].

## Data Availability

The data supporting this paper are held in a public repository, https://data.ncl.ac.uk/ (accessed on 2 July 2024).

## Supplementary Materials

The following supporting information can be downloaded at: https://www.mdpi.com/article/10.3390/jox14030050/s1, Figure S1: Xenotech HPCH05+ hepatocytes’ donor information and CYP probe substrate activities; Figure S2: M8OI MTT reduction in hepatocytes isolated from Liver A and B.

## Abbreviations

AcDH, acetaldehyde dehydrogenase; ADH, alcohol dehydrogenases; COOH7IM, 1-(7-carboxyheptyl)-3-methyl-1H-imidazol-3-ium; CPSI, carbamylphosphate synthase I; CPZ, chlorpromazine; CYP, cytochrome P450; HO8IM, 1-(8-Hydroxyoctyl)-3-methyl-imidazolium; M8OI, 1-octyl-3-methylimidazolium chloride, also known as C8mim; MILs, methylimidazolium ionic liquids; MTT, thiazolyl blue tetrazolium bromide; PBC, primary biliary cholangitis; POR, cytochrome P450 reductase.
