# Supplementary material for: Ionic Liquid 1-Octyl-3-Methylimidazolium (M8OI) Is Mono-Oxygenated by CYP3A4 and CYP3A5 in Adult Human Liver
Source: J Xenobiot. 2024 Jul 9;14(3):907–22. doi: 10.3390/jox14030050 (PMC11270251; doi:10.3390/jox14030050)
Supplement: Supplementary file 1 [file jox-14-00050-s001.zip › jox-2992090-supplementary.pdf]

XENOTECH
A BioIVT Company

Data Sheet

CryostaX

Single Freeze Pooled Plateable Cryopreserved Human Hepatocytes

HPCH05+ Lot No. 2110283

Pool of 5

Assured Minimum Yield:	5.0 x 10^6 cells per vial
Viability:	88%
Recommended Seeding Density	1.3 million cells per mL

Enzyme	***Marker Substrate Reaction***	***[S] (μM)***	***Rate (pmol/million cells/min)***
CYP1A2	Phenacetin *O*-dealkylation	100	10.7 ± 0.48
CYP2B6	Bupropion hydroxylation	500	9.37 ± 2.06
CYP2C8	Amodiaquine *N*-dealkylation	20	20.7 ± 1.6
CYP2C9	Tolbutamide hydroxylation	150	12.3 ± 0.95
CYP2C19	*S*-Mephenytoin 4'-hydroxylation	400	5.96 ± 0.12
CYP2D6	Dextromethorphan *O*-demethylation	75	15.4 ± 1.9
CYP3A4/5	Midazolam 1'-hydroxylation	30	23.8 ± 1.7
UGT	7-Hydroxycoumarin glucuronidation	100	233 ± 29
SULT	7-Hydroxycoumarin sulfonation	100	17.4 ± 1.9

Values for enzyme activities were determined at a single substrate concentration and are mean ± standard deviation of three or more determinations.

To measure cytochrome P450 (CYP), UDP-glucuronosyl transferase (UGT) and sulfotransferase (SULT) activities, cultured human hepatocytes (~ 20 hours post plating) were incubated in triplicate at 37 ± 2 °C for 30 or 45 minutes (substrate dependent) in OptIncubate and marker substrate, at the final concentrations indicated. Metabolite formation was determined by validated LC-MS/MS methods with deuterated metabolites as internal standards.

Donor Information

**Gender:**	Males (2), Female (3)
**Age:**	19-66 years of age
**Race:**	Caucasian (5)
**Cause of Death:**	Head trauma (3), Cerebrovascular accident (1), Anoxia (1)
**Antibody to Cytomegalovirus (CMV):**	Positive (3), Negative (2)
**Human Immunodeficiency Virus (HIV):**	Negative (5)
**Hepatitis B Surface Antigen (HbsAg):**	Negative (5)
**Antibody to Hepatitis C Virus (HCV):**	Negative (5)

Store in liquid nitrogen, vapor phase

CAUTION: This sample should be considered as a potential biohazard and universal precautions should be followed. Intended for *in vitro* use only.

These data were generated by and are the property of XenoTech. These data are not to be reproduced, published or distributed without the express written consent of XenoTech.

Datasheet prepared 28 October 2021

1101 W Cambridge Cir Dr | Kansas City, KS 66103 | p (877) 588-7530 / +913.438.7450 | f (913) 227-7100 | xenotech.com | info@xenotechllc.com

Figure S1. Xenotech HPCH05+ hepatocytes' donor information and CYP probe substrate activities

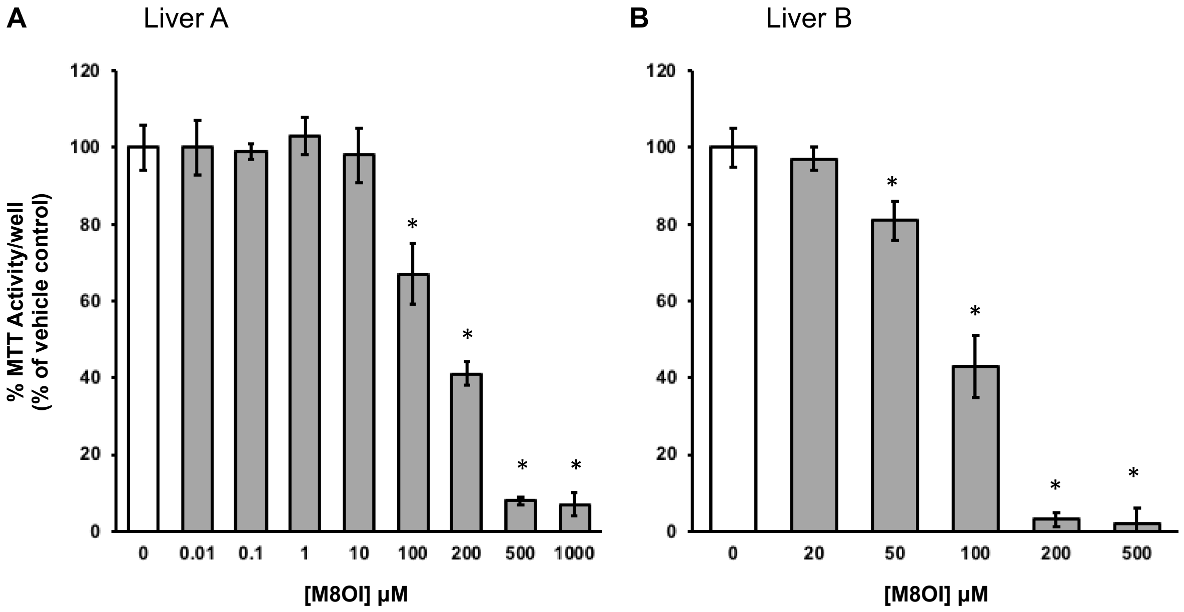

Figure S2. MTT activity in Liver A and Liver B hepatocytes.

Hepatocytes (Liver A (**A**); Liver B (**B**) were treated with increasing concentrations of M8OI for 24 hours in 24 well plates; control hepatocytes treated with 0.1% (v/v) DMSO vehicle. MTT reduction, data are the mean and SD of 4 separate determinations from the same donor. * P>0.95.
